# Engineering terpenoid production through transient expression in *Nicotiana benthamiana*

**DOI:** 10.1007/s00299-018-2296-3

**Published:** 2018-05-21

**Authors:** James Reed, Anne Osbourn

**Affiliations:** Department of Metabolic Biology, John Innes Centre, Norwich Research Park, Norwich, NR4 7UH UK

**Keywords:** Metabolic engineering, Agroinfiltration, Molecular diversity, Combinatorial biosynthesis, Natural products, Synthetic biology

## Abstract

Terpenoids are the most structurally diverse class of plant natural products with a huge range of commercial and medical applications. Exploiting this enormous potential has historically been hindered due to low levels of these compounds in their natural sources, making isolation difficult, while their structural complexity frequently makes synthetic chemistry approaches uneconomical. Engineering terpenoid biosynthesis in heterologous host production platforms provides a means to overcome these obstacles. In particular, plant-based production systems are attractive as they provide the compartmentalisation and cofactors necessary for the transfer of functional pathways from other plants. *Nicotiana benthamiana*, a wild relative of tobacco, has become increasingly popular as a heterologous expression platform for reconstituting plant natural product pathways, because it is amenable to *Agrobacterium-*mediated transient expression, a scalable and highly flexible process that enables rapid expression of genes and enzymes from other plant species. Here, we review recent work describing terpene production in *N. benthamiana*. We examine various strategies taken to engineer this host for increased production of the target metabolite. We also look at how transient expression can be utilised for rapid generation of molecular diversity, including new-to-nature products. Finally, we highlight current issues surrounding this expression platform and discuss the future directions and developments which will be needed to fully realise the potential of this system.

## Introduction

Plant natural products have long provided humanity with a source of flavourings, fragrances, dyes, cosmetics, and medicines. The plant kingdom remains a major source of important pharmaceuticals including painkillers and anti-cancer agents (Atanasov et al. 2015). However, accessing sufficient quantities of these compounds from the producing species frequently proves challenging due to low abundance of the target metabolite, slow growth or difficulty in propagation of the plant of origin and the challenges of purifying compounds from chemically complex plant extracts. Furthermore, the structural complexity of many plant natural products makes current methods of chemical synthesis unfeasible or uneconomical. As our understanding of plant natural product biosynthesis increases, expression of these pathways in heterologous hosts provides an opportunity to address these problems to meet the continued need for both currently used and new natural products.

**Fig. 1:**
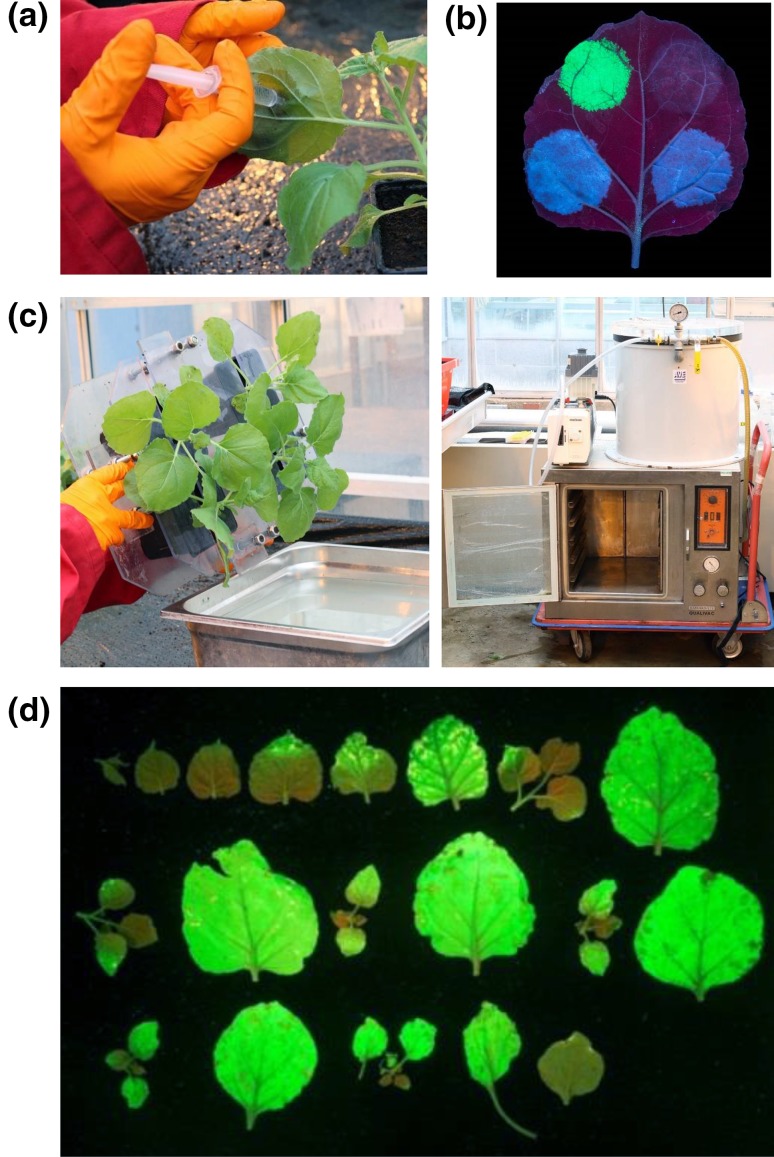
Transient expression in *N. benthamiana*. **a** Hand infiltration of *N. benthamiana* with a suspension of *A. tumefaciens* using a needle-less syringe. **b** Transient expression of protein and metabolites in *N. benthamiana*. Leaf viewed under UV 5 days after infiltration. Upper left—expression of green fluorescent protein. Bottom left and right—production of UV-fluorescent *N-*methyl anthranilate through expression of an oat methyltransferase and glycosyltransferase. Upper right—empty vector control. Picture courtesy of Aymeric Leveau and the Norwich Research Park (NRP) Image Library (**c**) Vacuum infiltration of *N. benthamiana*. Plants are inverted into a suspension of *A. tumefaciens* (left) and placed into a vacuum chamber (right). The vacuum is used to draw air from the interstitial leaf space, which is replaced by the inoculum upon release of the vacuum. **d** Leaves from a single *N. benthamiana* plant 5 days after vacuum infiltration with a GFP-carrying *A. tumefaciens*. Infiltration of most of the aerial parts of the plant is possible using this method. Leaves are arranged from top left to bottom right based on their height order (top–bottom) on the plant. This method provides much better coverage compared to infiltration by hand. Figure adapted from Reed et al. (2017)

*Nicotiana benthamiana*, a wild relative of tobacco, has seen increasing use over the past decade as a system for reconstituting plant natural product biosynthetic pathways. There are two main reasons underlying this. First, as a higher plant, *N. benthamiana* shares common cellular compartmentalisation, cofactors and coenzymes with other plants, thereby enabling the transfer of pathways from other plants without the need to extensively optimise the system. Second, this plant species is highly amenable to *Agrobacterium*-mediated transient expression, a process in which leaves are infiltrated using a needle-less syringe with a suspension of *Agrobacterium tumefaciens* carrying the gene(s) of interest within a binary vector; a process referred to as agroinfiltration (Fig. 1a). This approach is rapid, with gene expression and detection of the target protein or product typically achieved within a few days (Fig. 1b). This property also makes *N. benthamiana* a popular host for the production of proteins such as virus-like particles (VLPs), which have significant utility as vaccines (Marsian and Lomonossoff 2016). Agroinfiltration is a highly flexible process; expression of multiple genes can easily be achieved by simultaneous coinfiltration of *A. tumefaciens* strains, each containing different expression constructs. This approach thereby enables expression of multi-step pathways or combinatorial biosynthetic libraries, without the need to build multi-gene constructs (discussed further below). While the necessary metabolic precursors are often present in sufficient quantities in *N. benthamiana* for the heterologously expressed enzymes to function, it is also possible to infiltrate such precursors into the leaves (Lau and Sattely 2015; Miettinen et al. 2014). This may be useful for testing enzyme activity towards specific substrates, or where the necessary precursors cannot be produced in sufficient quantities *N. benthamiana*. Finally, agroinfiltration can be scaled to infiltrate large numbers of plants through vacuum infiltration (Fig. 1c/d).

**Fig. 2:**
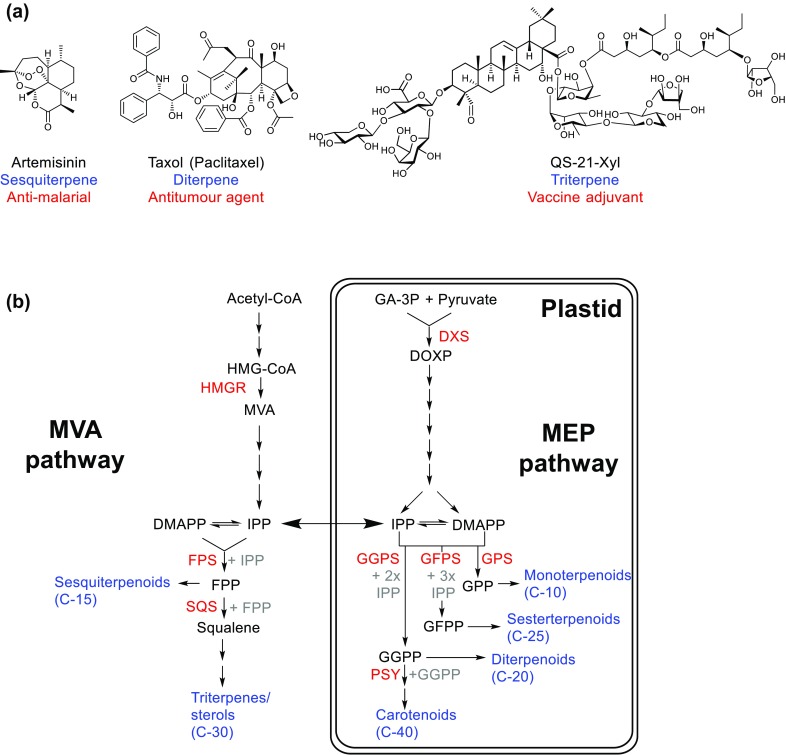
Terpene production in plants. **a** Examples of some medically important terpenes produced by plants. The names and class of the products are given in black and red, respectively, with the medical applications in blue. **b** Overview of the two terpene biosynthetic pathways in plant cells. Key biochemical intermediates are named in black, with important enzymes described in the main text named in red. The various classes of end products are named in blue. The MVA pathway is localised to the cytosol and associated endomembranes and is responsible for the production of sesquiterpenes and triterpenes. The MEP pathway is localised to the plastid and synthesises monoterpenes, diterpenes, sesterterpenes and carotenoids (tetraterpenes). *CoA* Coenzyme A; *HMG* 3-hydroxy, 3-methylglutaryl; *MVA* mevalonate; *IPP* isopentenyl diphosphate; *DMAPP* dimethylallyl diphosphate; *FPP* farnesyl diphosphate; *DOXP* 1-deoxy-d-xylulose 5-phosphate; *GPP* geranyl diphosphate; *GFPP* geranylfarnesyl diphosphate; *GGPP* geranylgeranyl diphosphate, *HMGR* HMG-CoA reductase; *FPS* FPP synthase; *SQS* Squalene synthase; *DXS* 1-deoxy-d-xylulose 5-phosphate synthase; *GPS* geranyl-diphosphate synthase; *GFPS* geranylfarnesyl-diphosphate synthase; *GGPS* geranylgeranyl-diphosphate synthase, *PSY* phytoene synthase

So far, pathways for several different types of natural products have been successfully reconstituted in *N. benthamiana*, including for alkaloids (Miettinen et al. 2014), lignans (Lau and Sattely 2015), cyanogens (Rajniak et al. 2015), betalains (Polturak et al. 2016), ketides (Andersen-Ranberg et al. 2017) and glucosinolates (Crocoll et al. 2016; Geu-Flores et al. 2009; Pfalz et al. 2011). These successes have demonstrated the potential of *N. benthamiana* for production of plant specialised metabolites. This review aims to highlight some key examples, focusing on terpene production. Terpenes (or isoprenoids) are the most structurally diverse class of plant specialised metabolites. They have a long historical use as flavourings and fragrances, but there are also numerous examples of high-value terpene therapeutics, such as the anti-cancer diterpene taxol, the anti-malarial sesquiterpene artemisinin and the triterpene vaccine adjuvant QS-21 (Fig. 2a). The difficulty of obtaining these compounds from their natural sources has driven the search for a sustainable and affordable supply. This in turn has prompted a large number of studies looking to engineer production of these and other compounds in heterologous hosts.

## Engineering terpene production in *N. benthamiana*

Most eukaryotes, including animals, fungi and higher plants share a common pathway for terpenoid biosynthesis, known as the mevalonate (MVA) pathway. However, in addition to the MVA pathway, plants possess a second, plastidial 2-*C*-methyl-d-erythritol 4-phosphate (MEP) pathway (Lichtenthaler et al. 1997). Both pathways produce common 5-carbon building blocks isopentenyl diphosphate (IPP) and its isomer dimethylallyl diphosphate (DMAPP), but are derived from different starting substrates (Fig. 2b). Although crosstalk between these pathways may occur (Bick and Lange 2003; Hemmerlin et al. 2003, 2012; Schuhr et al. 2003), the MEP pathway is generally considered responsible for production of monoterpenes (C-10), diterpenes (C-20) and tetraterpenes/carotenoids (C-40), while sesquiterpenes (C-15) and triterpenes (C-30) are synthesised via the MVA pathway (Vranova et al. 2013). This situation is conserved across higher plants, thus terpene biosynthetic enzymes derived from distantly related plant species generally seem to be correctly targeted and function appropriately in *N. benthamiana*. For example, sesterterpenes are a rare class of plastid-derived C-25 terpenes that are not naturally produced in *N. benthamiana*. Until recently, the terpene synthases that make these compounds (sesterterpene synthases) had not been characterised from plants. However, such enzymes have recently been identified in Brassicaceae species. Coexpression of various *A. thaliana* geranylfarnesyl diphosphate synthases (GFPS) with sesterterpene synthases allowed for production of various cyclised sesterterpenes in *N. benthamiana* (Huang et al. 2017b). In addition, the yield of the target pathway end product can often be enhanced by overcoming upstream pathway bottlenecks or countering the flow of intermediates into competing pathways. Hence, a good understanding of the pathway relevant to the metabolite of interest is key to successful engineering of the intended metabolite. Thus, significant attention has been given to understanding the important regulatory enzymes in each of the two pathways, and how they influence yield. Transient expression, therefore, provides a rapid method of screening candidate genes.

For the MVA pathway, 3-hydroxy-3-methylglutaryl-CoA reductase (HMGR) is known to be a key rate-limiting enzyme. The N-terminus of HMGR has an important regulatory function but is dispensable for catalytic activity. Truncation of this region results in a feedback-insensitive protein (tHMGR) and enhances yields of MVA end products such as squalene (Polakowski et al. 1998). This approach forms a standard strategy for enhancing production of MVA-derived sesquiterpenes and triterpenes (Cankar et al. 2015; Liu et al. 2014; Reed et al. 2017; van Herpen et al. 2010) (Table 1; Fig. 2). For MEP-derived mono- and diterpenes, expression of a prenyltransferase enzyme producing the relevant C-10 or C-20 prenyl diphosphate substrate can enhance yields (Dong et al. 2016; Yin et al. 2017). However, it may also be necessary to heterologously express the first committed MEP pathway enzyme, 1-deoxy-d-xylulose 5-phosphate synthase (DXS) to enhance the accumulation of end products (Andersen-Ranberg et al. 2016; Brückner and Tissier 2013) (Table 2; Fig. 2). The choice of plant species from which the yield-boosting enzymes are derived does not seem to be critical, for example the above studies utilised HMGR enzymes derived from oat or *A. thaliana* (Cankar et al. 2015; Liu et al. 2014; Reed et al. 2017; van Herpen et al. 2010), DXS from *Solanum lycopersicum* or *Coleus forskohlii* and prenyl transferases from peppermint, *C. forskohlii* and *Nicotiana tabacum*. At the same time, differences in relative activity can influence the extent to which an enzyme may be effective at boosting yields and testing multiple isoforms may be necessary (Dong et al. 2016).

**Table 1:** Examples of various MVA-derived terpenes produced through transient expression in *N. benthamiana*

Compound	Class	Quantity	Strategy	Fold increases	References
Linalool/Caryophyllene	Mono/Sesquiterpene	–	RNAi of *N. benthamiana* VAMP72 genes	Fivefold increase upon silencing VAMP72 genes	Ting et al. (2015)
Costunolide	Sesquiterpene	60 ng/g FW	Targeting to mitochondria	15-fold increases from mitochondrial-targeting versus cytosol	Liu et al. (2011)
Parthenolide	Sesquiterpene	1.4 µg/g FW	Expression of HMGR	Fourfold increases in the parthenolide precursor costunolide with HMGR	Liu et al. (2014)
(+)-Valencene	Sesquiterpene	0.70 µg/g FW/ 24 h (unopti-mised)	Expression of tHMGR & FPS, silencing of SQS and EAS	2.9-fold increases from expression of tHMGR and FPS	Cankar et al. (2015)
Artemisinic acid	Sesquiterpene	16.6 mg/kg FW	Genes fused together with use of 2A ribosomal skipping sequences/mitochondrial-targeting of ADS and FPS/ expression of tHMGR	Use of the fusion construct with mitochondrial FPS and tHMGR increased amorphadiene (artemisinic acid precursor) in headspace by ~ twofold, and internal leaf amorphadiene by ~ 7-fold	van Herpen et al. (2010)
Artemisinin	Sesquiterpene	3 ng/mg DW	Expression of LTP3 and PD2 plus HMGR	Approx 50% increase in artemisinin at 13 days after infiltration	Wang et al. (2016)
12,13-epoxy, 16-hydroxy-β-amyrin	Triterpene	**1.18 mg**/**g DW**	–	–	Geisler et al. (2013)
Various	Triterpene	**0.12–3.3 mg**/**g DW**	tHMGR, vacuum infiltration	Fourfold increases in β-amyrin upon expression of tHMGR	Reed et al. (2017)

An emphasis is placed on studies reporting either a specific yield or those which describe engineering approaches to improve yields of target compounds. Quantities are reported as given in the referenced study, these generally correspond to predicted yields from GC- or LC- quantification. Where isolated yields are reported, these are highlighted in bold. Where engineering strategies are described, the approximate fold increase in target compound is also given

*FW* Fresh weight, *DW* dry weight, *VAMP* vesicle-associated membrane protein, *(t)HMGR* HMG-CoA reductase (“t” denotes N-terminal-truncated form), *FPS* farnesyl diphosphate synthase, *ADS* amorphadiene synthase, *SQS* squalene synthase, *EAS* 5-*Epi*-aristolochene synthase, *LTP3* lipid transfer protein 3, *PD2* pleiotropic drug resistance 2

**Table 2:** Examples of various MEP-derived terpenes produced through transient expression in *N. benthamiana*

Compound	Class	Quantity	Strategy	Fold increases	References
Various	Monoterpene	5.55–19.08 µg/g FW/24 h	Expression of GPPS small subunit	~ 4–5 fold increases of (−)linalool	Yin et al. (2017)
Geraniol	Monoterpene	27 µg/g FW	–	–	Vasilev et al. (2014)
Geraniol	Monoterpene	93 µg/g FW	–	–	Fischer et al. (2013)
Geraniol	Monoterpene	129 µg/g FW	Targeting to different subcellular compartments (cytosol, mitochondria and plastid)	~ 2-3-fold increases with plastid-targeted GES and GPPS versus plastid-targeted GES alone	Dong et al. (2016)
18-Hydroxy dolabella-3,7-diene^a^	Diterpene	0.26 mg/g FW	Targeting to mitochondria	–	Dickschat et al. (2017)
Isopimaric acid	Diterpene	45–55 µg/g DW	Expression of DXS and GGPPS	3-fold	Gnanasekaran et al. (2015)
Taxadiene	Diterpene	48 µg/g DW	Silencing of native PSY	1.9-fold	Hasan et al. (2014)
Various	Diterpene	**0.5–5 mg**	Expression of DXS and GGPPS vacuum infiltration	~ 10-fold increases with combination of DXS and GGPPS	Andersen-Ranberg et al. (2016)
Cembratrienol	Diterpene	2500 ng/cm^2^	Coexpression of DXS and GGPPS	3.5-fold increase with DXS + GGPPS2	Brückner and Tissier (2013)
Various	Sesterterpene	**0.03–1.13 mg**/**g DW**	Vacuum infiltration	–	Huang et al. (2017b)
Various	Sesterterpene	**0.02–0.97 mg**/**g DW**	Expression of DXS, vacuum infiltration	–	Huang et al. (2017a)

An emphasis is placed on studies reporting either a specific yield or those which describe engineering approaches to improve yields of target compounds. Quantities are reported as given in the referenced study, these generally correspond to predicted yields from GC- or LC- quantification. Where isolated yields are reported, these are highlighted in bold. Where engineering strategies are described, the approximate fold increase in target compound is also given

*FW* Fresh weight, *DW* dry weight, *GPPS* geranyl-diphosphate synthase, *GGPPS* geranylgeranyl-diphosphate synthase, *GES* geraniol synthase, *DXS* 1-deoxy-d-xylulose 5-phosphate synthase, *PSY* phytoene synthase

^a^This is a bacterial diterpene synthase derived from *Chitinophaga pinensis*

Overexpression of these key enzymes is usually sufficient to enhance accumulation of the target compounds several-fold (Tables 1, 2). Nevertheless, expression or silencing of other enzymes at key branch points in the pathway has also been employed as a strategy to further enhance yields. For example, in the MVA pathway, a 2.8-fold increase in sesquiterpene (+)-valencene levels was achieved by simultaneous silencing of endogenous *N. benthamiana* squalene synthase (SQS) and sesquiterpene (5-*epi-*aristolochene) synthase which compete with the heterologous valencene synthase for available substrate (farnesyl diphosphate, FPP) (Cankar et al. 2015). SQS appears to be limiting for triterpene production, since transient expression of an oat SQS has been shown to enhance yields of β-amyrin by 2-3-fold (Reed et al. 2017). For the MEP pathway, Hasan et al. (2014) demonstrated that yields of taxadiene (the core scaffold for the diterpene anti-cancer agent taxol), could be enhanced in *N. benthamiana* through silencing of endogenous phytoene synthase (PSY), which competes with taxadiene synthase for geranylgeranyl diphosphate (GGPP). This approach was sufficient to enhance taxadiene levels almost twofold from 25 µg/g dry leaf weight (DW) in control lines to 48 µg/g DW (Hasan et al. 2014). Finally, improved yield of the target compound can also be achieved by targeting processes outside of the immediate biosynthetic pathway. For example, one interesting study employed agroinfiltration to silence *N. benthamiana* SNARE proteins (which mediate vesicular fusion) with simultaneous transient expression of linalool and caryophyllene synthases. This approach increased the levels of products by fivefold relative to non-silenced controls (Ting et al. 2015). Surprisingly this appeared to be an indirect effect, and was linked to reduced proteasome function, concordant with increased stability of the ectopically expressed terpene synthase proteins.

Other approaches to engineering terpene production in *N. benthamiana* have aimed to exploit the compartmentalisation of isoprenoid biosynthetic pathways in plants. By addition (or removal) of target peptides, the subcellular location of the transiently expressed protein can be altered in *N. benthamiana*, thereby deriving substrates from a different source to the native protein. For example, targeting sesquiterpene synthases to the mitochondria has been explored in several studies (Eljounaidi et al. 2014; Liu et al. 2011; van Herpen et al. 2010). In one case, this approach resulted in 15-fold higher accumulation of the target metabolite (costunolide) versus the native, cytosolic protein (Liu et al. 2011), suggesting that FPP may be more readily available in this compartment. Mitochondria do not appear to have a dedicated terpenoid biosynthesis pathway, relying on import of IPP from other compartments (Disch et al. 1998); therefore, the apparent reason behind the success of this strategy is poorly understood. In another example, transient expression was used to probe the capacity of chloroplasts, mitochondria and cytosol for biosynthesis of the monoterpene geraniol by systematic targeting of a geraniol synthase (GES) and geranyl-diphosphate synthase (GPS) to each compartment (Dong et al. 2016). This revealed that targeting to the plastids (the native compartment for GPS and GES) resulted in the highest levels of geraniol and derivatives, followed by mitochondrial and cytosolic targeting. Interestingly, in this study replacing the native GES plastid targeting peptide with an artificial version resulted in a moderate (1.2-fold) increase in geraniol (Dong et al. 2016). The results of this and similar studies clearly demonstrate that transient expression is an excellent means to rapidly probe various biosynthetic strategies for optimising target yields. Altering the subcellular compartments of terpene synthases has also been shown to be a valid strategy for increasing yields in transgenic plants (Farhi et al. 2011; Wu et al. 2006, 2012). Therefore, the findings of these studies may be used to inform strategies for regulating expression in stable lines.

## Rapid engineering of molecular diversity through transient expression

**Fig. 3:**
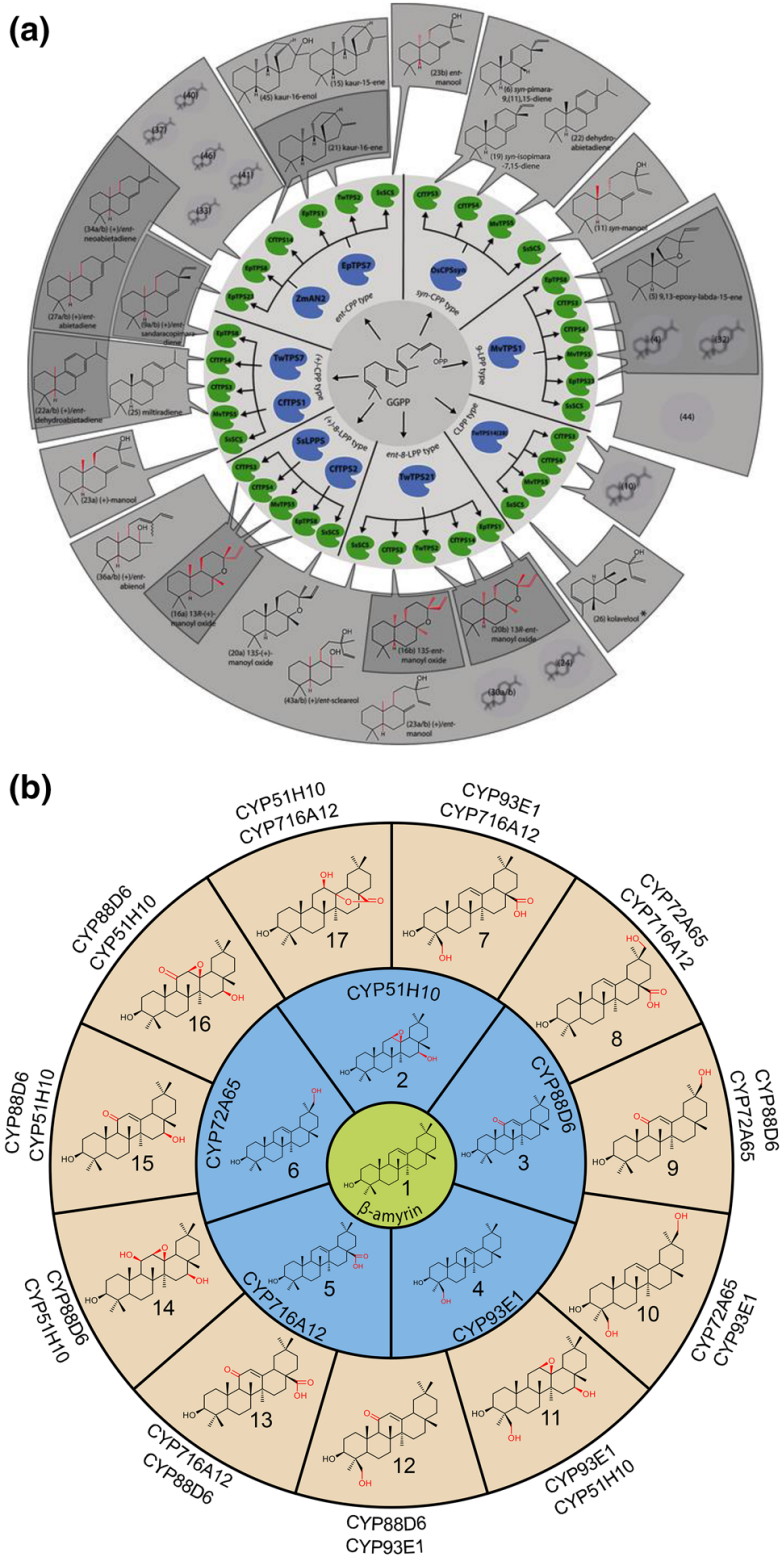
Combinatorial biosynthesis in *N. benthamiana*. **a** Production of diterpene scaffolds in *N. benthamiana* though combination of pairs of class II (inner circle, blue) and class I (inner circle, green) cyclases. Bonds in a specified stereochemical configuration formed through stereoselective controlled synthesis are highlighted in red. Bonds in a specified stereochemical configuration for which there is no biosynthetic access to the other configuration are shown in black. The **a** and **b** suffixes indicate (+) and ent configurations, respectively, as dictated by the class II diterpene synthases. Diterpenes with unknown structure are shaded in grey. *Clerodane diterpenes. **b** Production of oxygenated triterpenes. The products of individual P450s acting upon β-amyrin (centre) are shown in the inner blue circle. The major combinatorial products arising from pairs of P450s are shown in the outer circle. **a** adapted from Andersen-Ranberg et al. (2016), **b** adapted from Reed et al. (2017)

Enzymes in specialised metabolism often display activity towards non-native substrates, a phenomenon which can be exploited to access new molecular diversity. Coinfiltration of *A. tumefaciens* strains each containing different expression constructs provides a powerful means of rapidly expressing novel enzyme combinations, including enzymes from different plant species in a process frequently termed combinatorial biosynthesis. This type of approach has recently been demonstrated for accessing new diterpene scaffolds, which may be formed from GGDP in a two-step process performed by the combined action of discrete class II and class I cyclases (Zerbe and Bohlmann 2015). By employing systematic transient expression of combinations of 11 class II and 9 class I cyclases, Andersen-Ranberg et al. (2016) successfully obtained 51 functional pairings, resulting in a significant proportion of “new-to-nature” products with potential utility as new molecular scaffolds (Fig. 3a).

Combinatorial biosynthesis has also been exploited for diversification of terpene scaffolds. Much of the terpene structural diversity present in nature can be attributed to the action of tailoring enzymes, such as cytochrome P450s, glycosyl- and acyltransferases, which can produce thousands of variants from commonly occurring scaffolds (Hamberger and Bak 2013; Seki et al. 2015). The ubiquitous triterpene β-amyrin is one such example and derivatives of this molecule from different plants have a remarkable array of properties as sweeteners, anti-inflammatory-, anti-fungal- and adjuvant agents. However, an understanding of the importance of individual structural modifications with respect to activity are poorly understood, and the scaffolds are difficult to selectively modify using current chemical synthetic chemistry methods. Cytochrome P450s are capable of performing highly regio- and stereoselective oxidation of their substrates and hence provide an excellent resource for achieving structural diversification. As a proof of concept, transient coexpression of a β-amyrin synthase with individual β-amyrin-oxidising P450s and combinations of pairs of P450s was performed, resulting in production of a series of β-amyrin derivatives featuring the specific modifications introduced by the respective P450s (Fig. 3b) (Reed et al. 2017). Several of these products were not represented in natural product databases. A number of these products were purified from *N. benthamiana* in up to gram quantities allowing for the biological activity of the products to be explored. The ability to selectively oxidise the scaffolds in this manner could provide functional handles for further modification using other biosynthetic enzymes, or semisynthetic chemistry. The ability to coinfiltrate multiple *A. tumefaciens* strains allows the same vectors and selectable markers to be used for each gene, irrespective of how they will be ultimately expressed *in planta*. Performing this procedure is relatively simple and can be adopted by a researcher with relatively little molecular biology training.

Ultimately, the approaches above could be expanded to large-scale assembly and screening of combinatorial libraries for new molecules. However, at present, this approach is limited by the relatively small number of characterised genes, representing only a tiny fraction of the thousands of terpene biosynthetic pathways in nature. Discovery of new biosynthetic pathways will be facilitated by the increasing availability of sequence data from non-model and non-crop species thanks to projects such as the one thousand plants (1KP) database (Matasci et al. 2014), its successor ten thousand plants (10KP) project (Normile 2017), and the JGI Open Green Genomes initiative (https://jgi.doe.gov/csp-2018-leebens-mack-open-green-genomes-initiative/). Nevertheless, even the genomes of well-characterised model species such as *A. thaliana* encode a significant wealth of biosynthetic enzymes and pathways which are uncharacterised due to the products being present either at trace levels or under specialised conditions. It is now recognised that a number of diverse plant specialised metabolic pathways are arranged into ‘operon-like’ clusters, whereby the enzymes required for a biochemical pathway are encoded by a series of physically colocalised genes (Nutzmann et al. 2016). This provides an opportunity to discover novel biosynthetic pathways by developing algorithms to mine for the presence of such clusters (Medema and Osbourn 2016; Owen et al. 2017). Once identified, expression of these colocalised genes in *N. benthamiana* can be performed to determine their functional relevance. This approach has previously been applied for identifying terpenoid biosynthetic pathways from species including from *A. thaliana*, cucumber and *Lotus japonicus* (Boutanaev et al. 2015; Huang et al. 2017b; Krokida et al. 2013). Future work in this area will also benefit from the rapidly reducing cost of gene synthesis, which could ultimately lead to the ability to screen entire libraries of candidate genes for activity (Kosuri and Church 2014).

## Issues for the future

It is clear that *N. benthamiana* shows a great deal of promise for the production of terpenes and there are a growing number of examples, where this host has been used to obtain isolated yields of the target compounds in milligram to gram quantities (Tables 1, 2) (Andersen-Ranberg et al. 2016; Huang et al. 2017a, b; Reed et al. 2017). At the same time, one of the drawbacks to achieving this level of production is the matter of scale. For example, while infiltration of a single leaf by hand (Fig. 1a/b) is sufficient for most analytical work, translating this approach to infiltrate the volume of plants required for milligram-to-gram quantities of the target compound is laborious. To address this challenge, we and others have utilised vacuum infiltration of *N. benthamiana* (Andersen-Ranberg et al. 2016; Huang et al. 2017a, b; Reed et al. 2017). This process involves inverting the aerial parts of *N. benthamiana* plants into a bath of *A. tumefaciens* and using a vacuum to draw the air from the interstitial spaces between the leaf cells (Reed et al. 2017). Upon equilibration to atmospheric pressure, the bacterial suspension is drawn into the leaves. This process allows for simultaneous batchwise infiltration of multiple plants at once. This process has been applied on a commercial scale for the production of pharmaceutical proteins in *N. benthamiana* (Holtz et al. 2015). For the academic lab, such devices can be constructed relatively cheaply with commercially available parts.

One of the issues that has confounded production of some molecules has been the observation of unintended side products resulting from modification of the target compound by endogenous enzymes, resulting in oxidation, glycosylation or dephosphorylation (Brückner and Tissier 2013; Dong et al. 2016; Khakimov et al. 2015; Liu et al. 2011; Wang et al. 2016). Although such reactions may give rise to new bioactive molecules (Liu et al. 2014), such crosstalk with the host metabolism is generally undesirable. Genome editing approaches (Li et al. 2013; Nekrasov et al. 2013) might, therefore, be employed to limit this phenomenon by knocking out endogenous sugar transferases or oxidases which crosstalk with the heterologously expressed pathway. Alternatively, approaches to minimise the potential for crosstalk with endogenous enzymes may be utilised to circumvent this problem. For example, transient expression of the Lipid Transfer Protein 3 (LTP3) and Pleiotropic Drug Resistance Transporter 2 (PD2) from *Artemisia annua* in *N. benthamiana* resulted in increased transport of artemisinin precursors to the apoplast, thereby reducing loss of intermediates to glycosylation by cytosolic endogenous glycosyltransferases (Wang et al. 2016).

Although the ability to infiltrate multiple strains offers a rapid way to assemble new gene combinations, the density of the bacterial inoculum must be increased with coinfiltration of additional *A. tumefaciens* cultures, which imposes practical limitations on the maximum number of different constructs that can be coexpressed (Montague et al. 2011). Therefore, integration of multiple genes into a single T-DNA provides a solution to this problem. One possible approach to this is to fuse multiple genes into a single open reading frame, separated by a viral peptide signal (2A) which results in ribosomal skipping and production of the individual proteins (van Herpen et al. 2010). An additional drawback of coinfiltration is the lack of fine control over the stoichiometry of the various coexpressed proteins due to differences in the efficiency of T-DNA delivery from different *A. tumefaciens* strains to individual leaf cells (Montague et al. 2011). Control over protein stoichiometry during transient expression has previously been demonstrated by modulating translation efficiency of the expressed genes in a single T-DNA with different 5′ UTR leader sequences. This approach allowed optimised production of Bluetongue virus VLPs in *N. benthamiana* (Thuenemann et al. 2013). For metabolic engineering, such an approach might be applied for controlling the stoichiometry of subunits of heteromeric enzymes, balancing enzymes and their respective coenzymes (such as cytochrome P450s and reductases) or optimising multiple steps in a pathway to balance flux through a biosynthetic pathway. Similarly, this could be achieved through use of synthetic promoters designed to have various strengths and which are controlled by a single orthogonal transcription factor. This approach has been demonstrated for engineering diterpene production levels upon transient expression in *N. benthamiana* (Brückner et al. 2015). Modular cloning techniques such as the type II restriction enzyme-based Golden Gate system (Weber et al. 2011) enable the researcher to choose from a library of validated parts including promoters, terminators and UTRs, thereby allowing fine tuning over expression of individual proteins (Engler et al. 2014).

## Conclusions and perspectives

Utilising heterologous host production systems has immense potential for accessing the thousands of bioactive plant specialised metabolites present in nature. Microbial production systems have demonstrated the potential of this approach to meet demand for high-value plant chemicals. The successful engineering of *Saccharomyces cerevisiae* for production of artemisinin serves as a flagship example (Paddon and Keasling 2014; Paddon et al. 2013). Other recent examples have also emerged of engineering this host to produce other complex high-value alkaloids (Brown et al. 2015; Galanie et al. 2015; Li and Smolke 2016). Nevertheless, these efforts have required extensive optimisation of the genetic background of the host strain in addition to expression of the biosynthetic pathway of interest to subvert yeast metabolism towards classes of products it has not evolved to produce. Furthermore, the yields of the end products are still in the microgram per litre range in many cases, thus highlighting the need to continue to explore other host systems. The numbers of studies demonstrating the purification of compounds derived from transient expression in plants have increased in recent years, reflecting the increasing popularity of this system (Andersen-Ranberg et al. 2016; Dickschat et al. 2017; Geisler et al. 2013; Huang et al. 2017a, b; Reed et al. 2017). As described above, transient expression can be achieved at industrial scale, and is currently used for production of pharmaceutical proteins by various companies (Sack et al. 2015). The linear nature of scaling vacuum infiltration is important for translating small scale experiments from an academic lab to commercial setting. This scalability, coupled with the rapid turnaround time of transient expression, allows vaccines to be produced within a matter of weeks and mobilised in response to emerging epidemics (Marsian and Lomonossof 2016; Owen et al. 2017). This principle could similarly be applied for on-demand access to small molecules. With this in mind, transient plant expression has the potential to be a disruptive technology for metabolic engineering of plant bioactives and other high-value chemicals.

### Author contribution statement

JR and AO co-wrote the manuscript.
